# Supplementary material for: A Global Screen for Assembly State Changes of the Mitotic Proteome by SEC-SWATH-MS
Source: Cell Syst. 2020 Feb 26;10(2):133–155.e6. doi: 10.1016/j.cels.2020.01.001 (PMC7042714; doi:10.1016/j.cels.2020.01.001)

Q9Y3B4 | SF3B6_HUMAN | SF3B6 SAP14 SF3B14 SF3B14A CGI−110 HSPC175 HT006
Monomer MW [kDa]: 14.585 Monomer expected elution fraction: 57

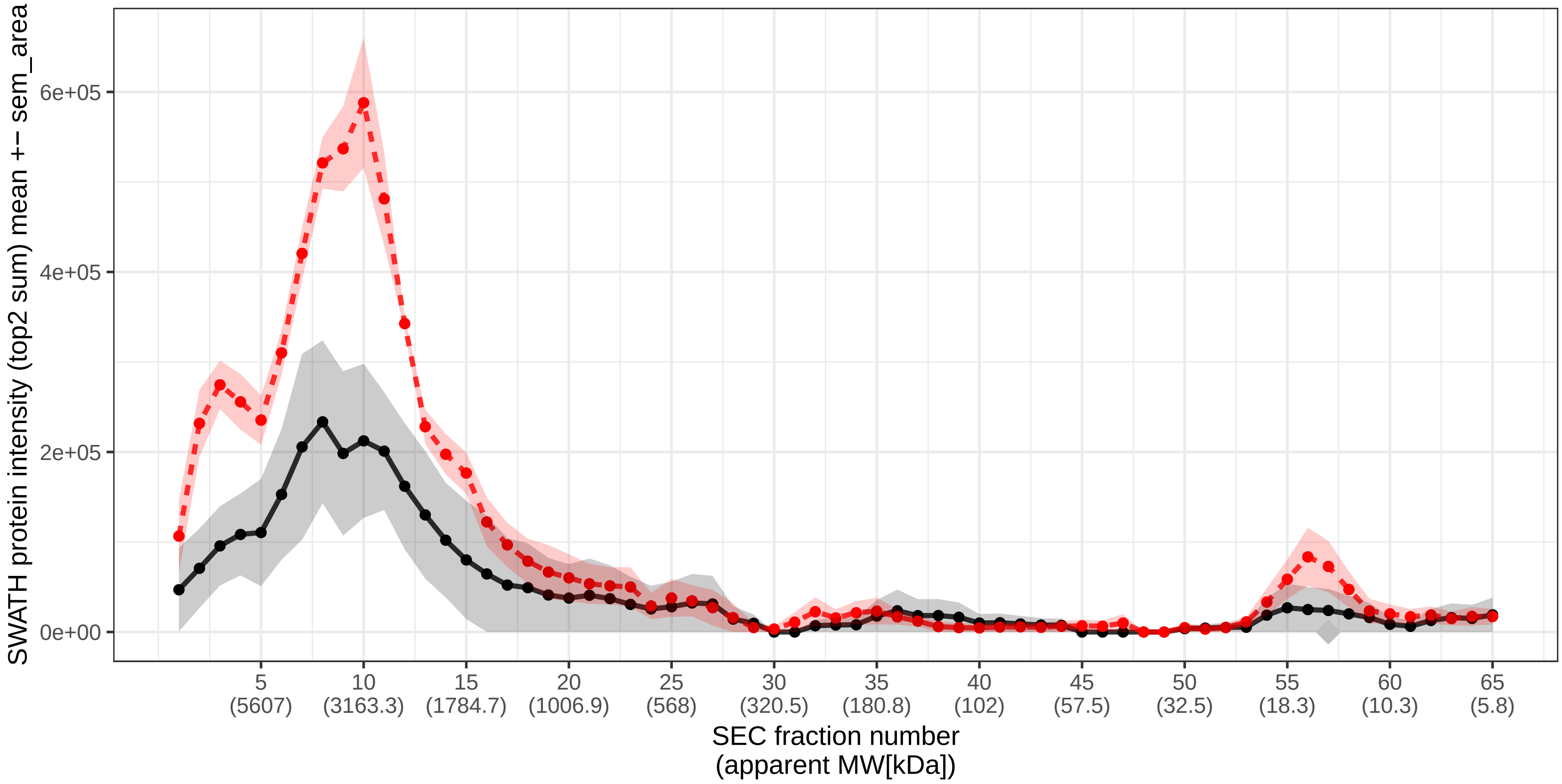

Supplement: Data S1. SEC-SWATH-MS Protein Chromatograms, Related to Figure 1 [file mmc6.zip › SECchrom_Q9Y3B4_SF3B6_HUMAN_SF3B6_SAP14_SF3B14_SF3B14A_CGI-.pdf]
